# Family-school-hospital collaboration model for urinary system tumor early screening education

**DOI:** 10.3389/fpubh.2026.1837667

**Published:** 2026-08-26

**Authors:** Fen Zhou, Ping Yu, Xiaofen Yu, Xuyan Han, Zheng Wang

**Affiliations:** Hangzhou Medical College, The Affiliated People’s Hospital of Hangzhou Medical College, Zhejiang Provincial People’s Hospital, Hangzhou, China

**Keywords:** 3D printing, core competencies, family WeChat group, family-school-hospital collaboration, medical students, public health, tumor early screening, tumor secondary prevention

## Abstract

**Objective:**

To develop and validate a 3D printing-assisted family-school-hospital collaborative education model for urinary system tumor early screening, and to evaluate its association with changes in medical students’ knowledge and core competencies related to tumor early detection.

**Methods:**

A non-randomized controlled pre-post study was conducted among 120 clinical medical interns (60 in each group). The intervention group received a 6-month family-school-hospital collaborative program via family WeChat groups, while the control group received traditional clinical teaching only. The primary outcome was knowledge score using questions from the National Medical Licensing Examination. Secondary outcomes included five core competencies: social medical curiosity, self-directed learning ability, doctor-patient communication ability, inter-professional teamwork competence, and transformative learning readiness. Data were analyzed using independent t-tests, change-score analysis.

**Results:**

After adjusting for baseline, the intervention group showed significantly greater improvements in knowledge score (mean change: 12.48 ± 9.98 vs. 6.28 ± 11.55; *p* = 0.0046, Cohen’s d = 0.56, 95% CI: 1.97 to 10.43), social medical curiosity (mean change: 1.55 ± 5.26 vs. 1.10 ± 5.02; *p* = 0.0458, Cohen’s d = 0.37, 95% CI: 0.037 to 3.896), self-directed learning ability (*p* = 0.0458, Cohen’s d = 0.38, 95% CI: 0.037 to 3.896), and doctor-patient communication ability (*p* = 0.0238, Cohen’s d = 0.39, 95% CI: 0.0494 to 0.6834). Inter-professional teamwork competence and transformative learning readiness showed no significant differences after baseline adjustment (both *p* > 0.05).

**Conclusion:**

The 3D printing-assisted family-school-hospital collaborative education model was associated with significant improvements in medical students’ knowledge and core competencies for urinary system tumor early screening. This localized, family-oriented approach provides a feasible and replicable teaching strategy for cancer early detection education, supporting the integration of medical education and public health practice.

## 1 Introduction

The core of tumor secondary prevention lies in early detection. For most malignant tumors such as lung cancer, breast cancer and urinary system tumors, there are significant differences in treatment outcomes, patients’ quality of life and social medical burden between early and late diagnosis. Promoting early tumor screening and diagnosis is a key measure to improve the effect of tumor prevention and control worldwide (1, 2). In China, community health workers have long focused on the management of chronic diseases such as hypertension and diabetes, and generally lack systematic oncology professional training, making it difficult to meet the core needs of primary-level comprehensive tumor secondary prevention (3). In the clinical practice of our team, it is also found that a large number of tumor patients have developed to an advanced stage when referred by interns and specialists, which highlights the urgency of activating professional medical forces at the family level and carrying out tumor early screening interventions.

At present, every extended family in China has a family WeChat group. Relying on universal coverage, this carrier realizes barrier-free participation of all ages, regions and cultural levels. The daily usage rate of WeChat has reached 97% (4), forming characteristics of high-frequency and real-time family communication, which provides a convenient and efficient digital platform for interns to spread knowledge of tumor early screening and promote community tumor secondary prevention under the family-school-hospital collaborative model. Meanwhile, Chinese people have unique natural trust among family members: many patients are unwilling to mention disease privacy (such as hematuria symptoms of urinary system tumors) to doctors and outsiders, but are willing to consult and talk to relatives in family WeChat groups (3). The groups generally include clinical medical students and medical workers of various majors who have graduated. Such a carrier has unique advantages for family-centered tumor intervention, yet it remains underutilized in public health and medical education. However, due to the disconnection between traditional medical education and public health needs, relevant application studies are scarce, and few studies have integrated family-school-hospital collaboration into malignant tumor secondary prevention.

As the smallest unit of public health intervention, the family is a key carrier for the implementation of tumor secondary prevention. Family-based health interventions can significantly improve the compliance of population preventive behaviors and health literacy (5, 6), and are also an important implementation carrier for secondary prevention of various diseases (7, 8). As a new medical teaching technology, 3D printing visualization models can highly faithfully restore the anatomical morphology of organs and pathological characteristics of early tumors, effectively make up for the shortcomings of traditional two-dimensional teaching, significantly improve medical students’ efficiency of anatomical cognition and clinical reasoning ability (9–12), and provide technical support for medical students to master tumor early screening knowledge (13, 14). The family-school-hospital collaborative platform integrates hospital, university and family resources, which is highly consistent with the public health concept of “multi-sector collaboration” (15–18). It can be implemented based on family WeChat groups, allowing medical students to learn combined with the real health cases of relatives under the guidance of tutors, and realize the transformation of clinical knowledge into family health guidance ability in the era of mobile Internet (19).

In view of the practical considerations of our team’s research strength and practice promotion, this study selects urinary system tumors as the entry point for comprehensive tumor early screening research. Urinary system tumors (renal cancer, bladder cancer, etc.) have a high incidence in both male and female populations. Due to the particularity of the disease site, patients often experience cultural stigma, and core early warning signs such as painless hematuria are frequently overlooked or concealed (3), which has become a key obstacle to early diagnosis and treatment. This clinical challenge makes urinary system tumors an ideal model to verify the effectiveness of the family WeChat group–based intervention for improving medical students’ core competencies in tumor early detection.

This study proposes and defines the general core competencies in tumor early detection as a comprehensive framework for family-level tumor early screening, including social medical curiosity, self-directed learning ability, doctor-patient communication, inter-professional teamwork competence, and transformative learning readiness. Social medical curiosity serves as the cognitive basis for identifying early abnormal symptoms among family members; self-directed learning ability ensures the mastery of screening knowledge and indications; inter-professional teamwork competence supports multidisciplinary collaboration; transformative learning readiness bridges clinical knowledge and family health practice; and doctor-patient communication helps reduce stigma and encourage screening. Based on China’s social context and comprehensive cancer control needs, this study developed a family-school-hospital collaborative teaching model to systematically cultivate medical students’ core competencies in tumor early detection and to strengthen family-based professional health resources. To our knowledge, no prior study has established this integrated model for urinary system tumor early screening education. This study aims to provide evidence for improving early diagnosis, establish a replicable family-centered screening framework, and support the integration of medical education and public health practice.

## 2 Materials and methods

**Figure 1:**
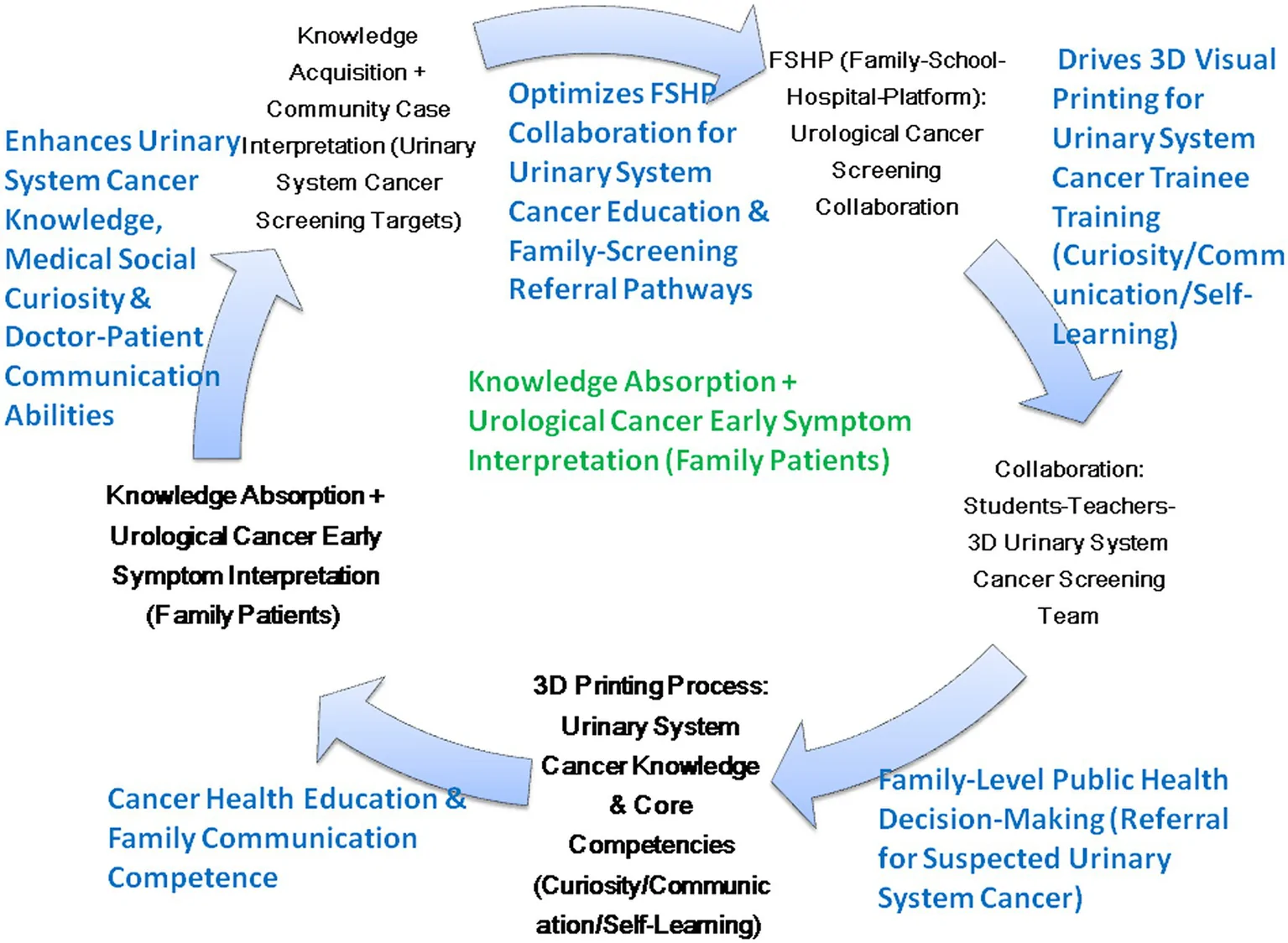
Conceptual framework of the 3D printing-assisted family-school-hospital collaboration model for early screening of urinary system cancer. This framework links 3D visual training, multi-stakeholder collaboration, and family-level health decision-making.

As illustrated in Figure 1, our intervention is based on a 3D printing-assisted family-school-hospital collaborative framework for early screening of urinary system cancer.

### 2.1 Study design and participants

This was a non-randomized controlled pre-post quasi-experimental study carried out in a provincial hospital of China from September 2025 to February 2026. A total of 132 undergraduate clinical medicine interns were enrolled in this research. Restricted by the arrangement and daily management of clinical internship, participants were divided into observation group and control group via non-randomized convenience grouping, with 66 individuals allocated to each group at the beginning.

Ultimately, 120 participants finished the whole research procedure, with 60 cases in each group. Twelve participants dropped out due to incomplete data submission and suspended internship, corresponding to an overall attrition rate of 9.09%. Among them, 6 participants withdrew from the observation group, including 3 cases with incomplete data and 3 cases with interrupted internship. Another 6 participants dropped out from the control group, consisting of 4 cases with incomplete data and 2 cases with suspended internship. No significant difference was detected in attrition rate and withdrawal reasons between the two groups (*χ*^2^ = 0.21, *p* = 0.647), which indicated negligible attrition bias in this research.

To reduce selection bias and guarantee group comparability, we conducted comprehensive baseline balance assessment. Demographic information including age, gender, ethnic background, only-child status and academic GPA, together with all primary and secondary outcome indicators were compared between groups. Here, academic GPA represents the unified national pre-internship standardized graduation examination score for clinical medical undergraduates across China, a mandatory nationwide assessment that objectively reflects students’ pre-clinical basic medical knowledge reserve. Detailed baseline results are displayed in Table 1, and no obvious inter-group differences were observed. All statistical analyses in this study adopted complete-case analysis. Only participants who fully completed the whole intervention and submitted all valid questionnaire data were included in the final comparative analysis.

**Table 1:** Baseline characteristics of participants in the control and observation groups.

Parameters	Traditional teaching group (*n* = 60)	FSHP+3D PVM teaching group (*n* = 60)	Test statistic (*t*/*χ*^2^)	*p* value
Age (years)	22.14 ± 0.86	22.43 ± 0.93	1.773	0.079
Gender (M/F)	31(51.67%/29(48.33%)	32(53.33%)/28(51.67%)	0.033	0.855
Ethnicity (Han)	54 (90.00%)	55(91.67%)	0.100	0.752
Residence (Urban/Rural))	38(63.33%)/22(36.67%)	36(60.00%)/24(40.00%)	1.410	0.235
Only-child (Yes)	30 (50.00%)	33 (55.00%)	0.301	0.582
Undergraduate GPA	4.97 ± 0.94	5.01 ± 0.96	0.231	0.818

Continuous variables are presented as mean ± standard deviation and compared using independent samples *t*-test; categorical variables are presented as *n* (%) and compared using Pearson’s chi-square test.

We further conducted sensitivity analysis to compare the baseline demographic and outcome indicators between 12 drop-out students and retained subjects, and no significant inter-group differences were observed (all *P*>0.05), which indicated that attrition bias was negligible in this research.

Sample size estimation was conducted using SPSS 23.0 software. Calculation parameters were set as follows: standard deviation of 3.5, inter-group mean difference of 2.1, type I error *α* = 0.05 and type II error *β* = 0.2. The minimal required sample size for each group was calculated as 60 participants. To maintain sufficient statistical power against potential participant loss and missing data, we expanded the initial sample size by 10%.

Inclusion criteria: ① Age no less than 21 years old; ② Completed all undergraduate theoretical courses and engaged in clinical graduation internship; ③ Fully acknowledged the research purpose, procedures and potential risks, and signed written informed consent voluntarily.

Exclusion criteria: ① Internship interrupted on account of personal, family or other special matters during the research; ② Continuous leave over 3 days or accumulated leave exceeding 7 days in the intervention stage, resulting in incomplete intervention experience; ③ Attended other similar medical teaching or tumor early screening training programs simultaneously; ④ Failed to finish questionnaires and knowledge tests as required, leading to incomplete or invalid research data.

This study adopted non-randomized convenience grouping according to the dormitory allocation rules of two campuses, which was restricted by fixed campus dormitory capacity. Random redistribution of accommodation was ethically infeasible, This grouping arrangement fully follows educational ethics equity principles: boarding interns have unified off-hours centralized learning space and time to participate in supplementary 3D model training and family WeChat group teaching activities. Random reallocation would force some trainees with available learning time to lose access to this optimized targeted tumor screening training resource, which violates the principle of fair access to educational resources. Commuting interns lack stable after-class collective learning conditions and thus were assigned to the conventional teaching control group voluntarily after full informed consent. as it would deprive some off-campus interns of standardized on-campus teaching resources. Beyond the baseline indicators listed in Table 1, we further compared potential confounding factors including family socioeconomic status, parents’ occupation, daily self-study time and pre-intervention learning motivation scores between two groups. No statistically significant inter-group differences were detected in these covariates (all *P*>0.05). The only inherent gap between groups was unified daily centralized learning time for boarding students.

### 2.2 Data collection methods

Data were collected via questionnaires, self-assessment reports and knowledge tests to comprehensively evaluate the effects of the 3D printing visual model-assisted family-school-hospital collaborative teaching mode. We measured interns’ mastery of National Medical Licensing Examination-related knowledge, social medical curiosity, self-directed learning ability, doctor-patient communication ability, inter-professional teamwork competence and transformative learning readiness.

Assessments were conducted at baseline (before the intervention) and upon completion of the 6-month teaching intervention (February 2026). All outcome evaluators were blinded to group allocation throughout the study. We strictly guaranteed data accuracy, protected participants’ privacy and minimized potential bias and errors.

### 2.3 Teaching intervention

The intervention was implemented from September 2025 to February 2026, lasting for 6 consecutive months.

The control group received traditional internship teaching in accordance with the Clinical Practice Manual, including routine bedside teaching, case discussion and theoretical teaching focusing on the diagnosis and treatment of urinary system diseases. No training on tumor early screening, 3D printing or WeChat group practice was arranged.

The observation group adopted a teaching model combining 3D printing assistance and family-school-hospital collaboration via the WeChat platform. Teaching was delivered offline and online, focusing on urinary anatomy, tumor screening guidelines and the application of 3D visual models. Weekly online group activities included health education, case discussion and remote tutor guidance. A closed-loop teaching system was established to help intern’s master relevant professional knowledge and practical skills (Figure 1).

### 2.4 Platform and model construction

We collected anatomical and imaging data of urinary organs and completed 3D reconstruction using professional medical digital software. The team held regular discussions to optimize model design to meet teaching demands.

**Figure 2:**
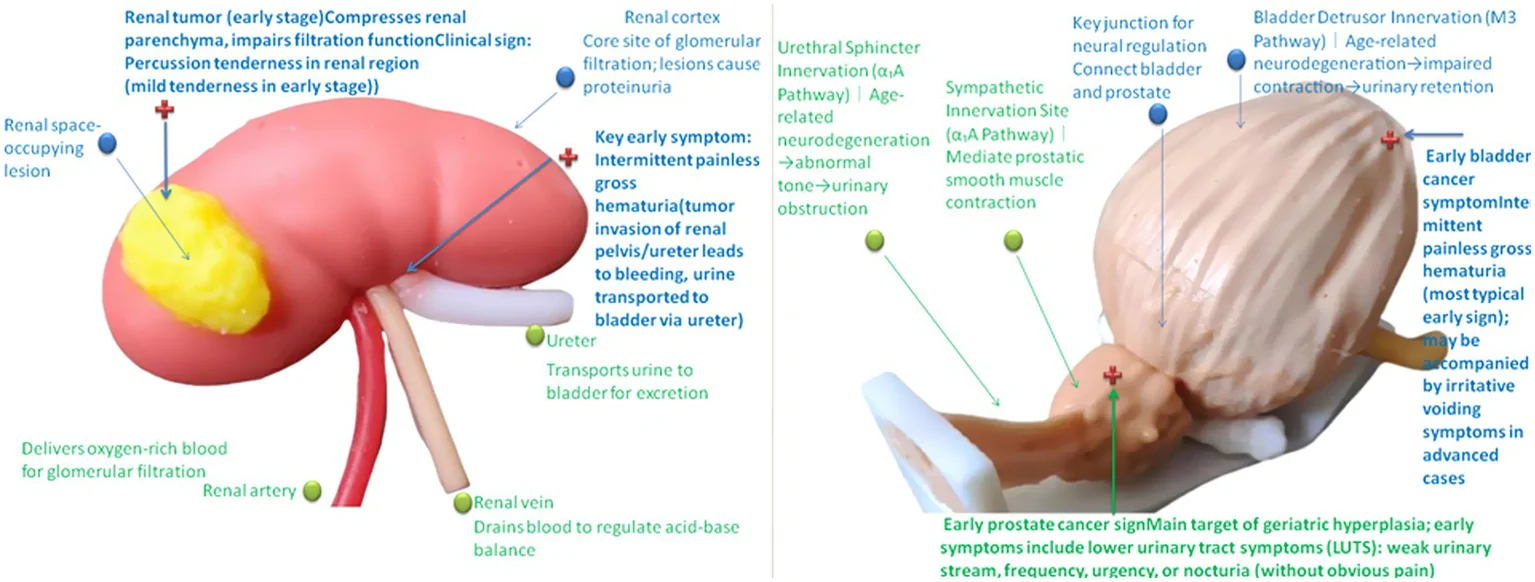
3D‑printed urinary system tumor teaching model used in the educational intervention.

Standard 3D printing techniques were used to produce organ models with anatomical and pathological characteristics. The models were colored and marked with key teaching points (Figure 2). Detailed specifications of software and printing devices are omitted in the main text.

### 2.5 Assessment tools

Primary outcome indicator: A 100-point theoretical knowledge test focusing on urinary system diseases and tumor secondary prevention. All items were selected from real questions of the National Medical Licensing Examination. As a nationally standardized assessment tool with verified quality, it was used to evaluate interns’ professional knowledge mastery.

Secondary outcome indicators:

1. Social Medical Curiosity (SMC): The Chinese version of the Medical Curiosity Scale translated by Yang et al. (20). Only the SMC subscale was adopted (Cronbach’s *α* = 0.866), which has good reliability and validity among Chinese medical students.
2. Self-directed Learning Ability (SDLA): The scale developed by Wang et al. (21), containing 4 dimensions and 28 items with a 5-point Likert scale (overall Cronbach’s α = 0.893), with solid validation in medical students.
3. Doctor-patient communication ability: The localized SEGUE Framework by Shen et al. (22), including 25 binary-scoring items covering five communication stages (total score: 0–25).
4. Inter-professional Teamwork Competence (AITCS): The simplified Chinese version by Cui et al. (23), consisting of 3 dimensions and 23 items (overall Cronbach’s *α* = 0.912).
5. Transformative Learning Readiness (MSTLR): The scale developed by He et al. (24), with 4 dimensions and 25 items using a 5-point Likert scale (overall Cronbach’s *α* = 0.887).

All scales are widely applied in Chinese medical education research, and their assessment contents match the research objectives of this study. After calculating the internal consistency of each scale based on the 120 medical interns in this study, all Cronbach’s *α* values of the scales and their sub-dimensions fell within the acceptable reliable range (α>0.80), which further proved that these measurement tools have stable and consistent measurement performance when applied to the subjects of this research.

### 2.6 Ethical approval

This study was approved by the Ethics Review Committee of Hangzhou Medical College (Approval No.: LL2025-011) and the Ethics Committee of Zhejiang Provincial People’s Hospital (Approval No.: 2025–488). All procedures involving human research participants were strictly conducted in accordance with the ethical principles of the Declaration of Helsinki (2024 version) of the World Medical Association. All research participants provided written informed consent, and data were collected anonymously.

## 3 Results

### 3.1 Baseline characteristics

No significant differences were found between the two groups in baseline characteristics (age, gender, ethnicity, only-child status, GPA) and all primary and secondary outcome indicators (all *p* > 0.05), indicating comparable baseline conditions of the two groups (Table 1).

### 3.2 Comparison of outcome indicators before and after intervention

**Figure 3:**
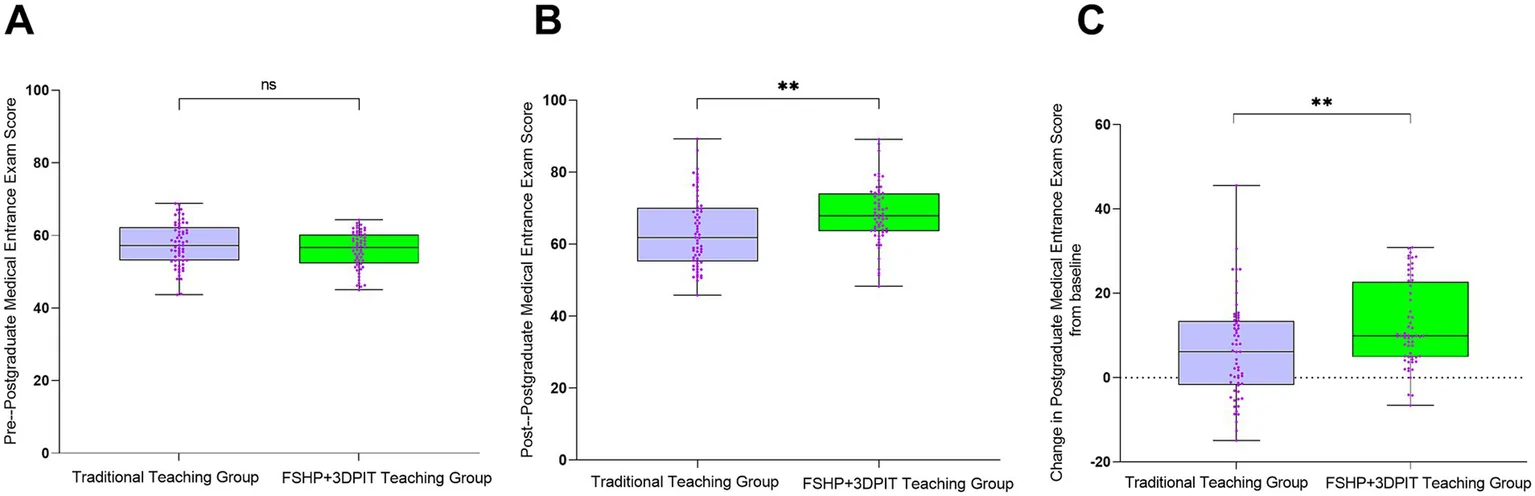
Box plots of NMLE simulation scores at baseline **(A)**, post‑intervention **(B)**, and score changes from baseline **(C)** in the two groups.

**Figure 4:**
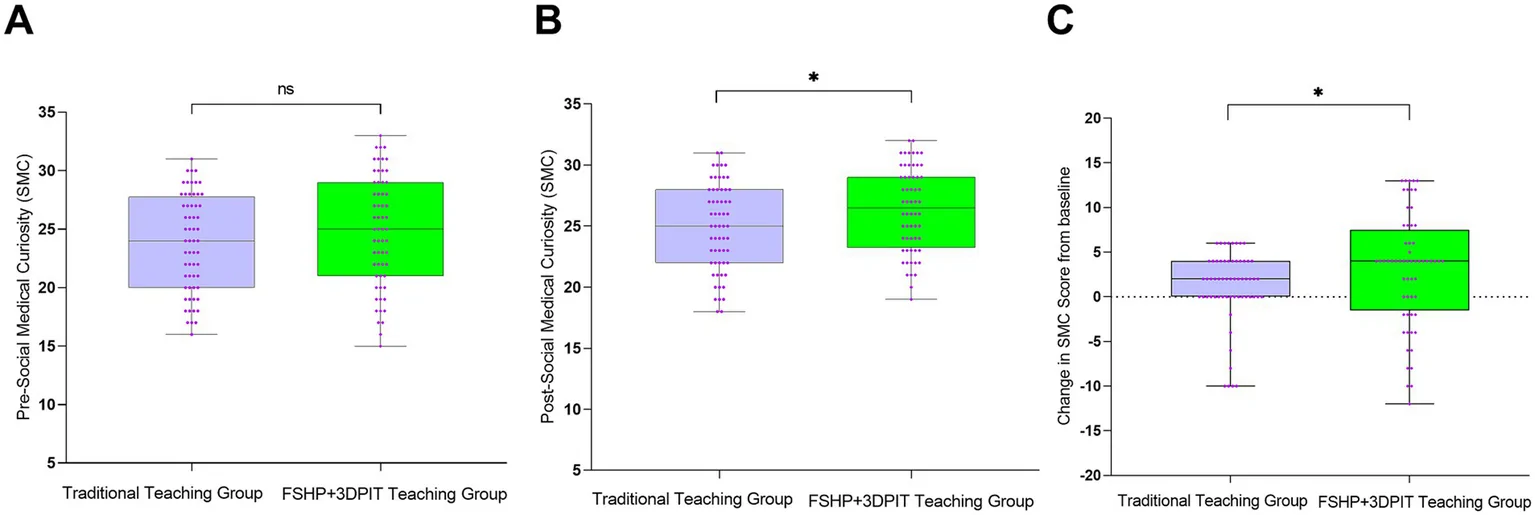
Box plots of SMC scores at baseline **(A)**, post‑intervention **(B)**, and score changes from baseline **(C)** in the two groups.

**Figure 5:**
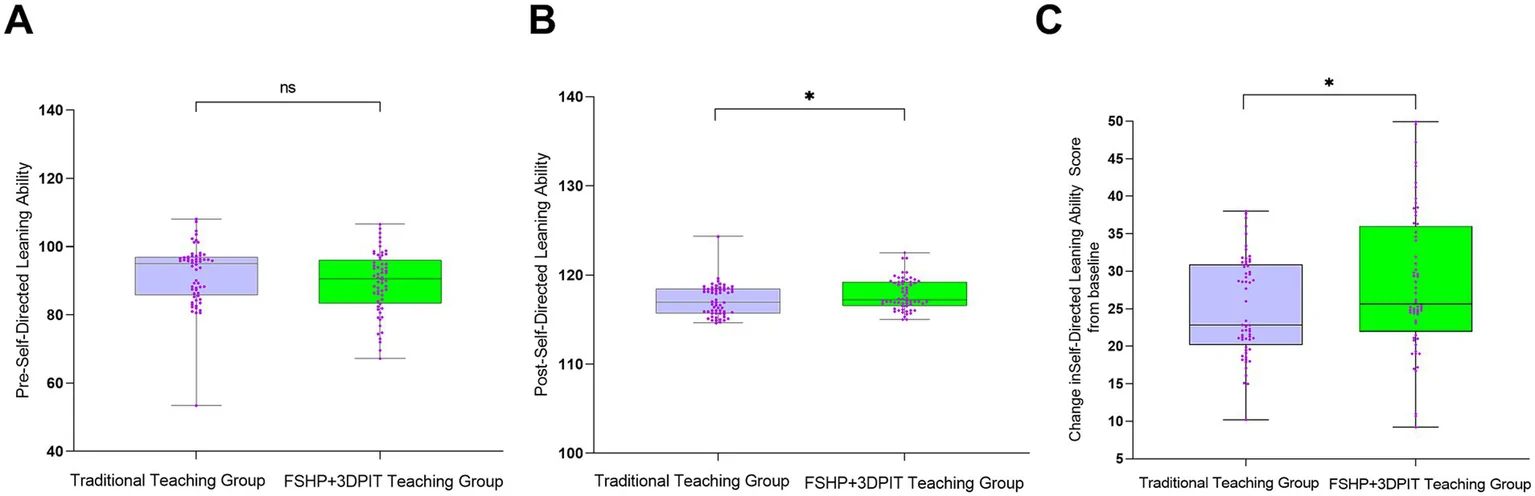
Box plots of SDLA scores at baseline **(A)**, post‑intervention **(B)**, and score changes from baseline **(C)** in the two groups.

**Figure 6:**
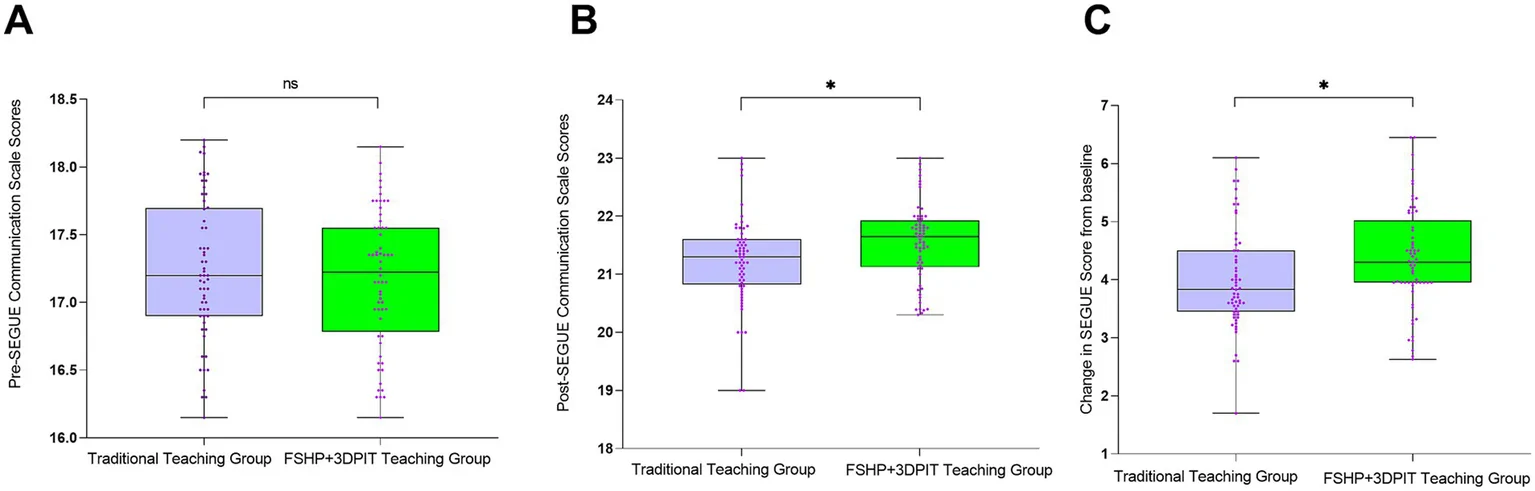
Box plots of SEGUE scores at baseline **(A)**, post‑intervention **(B)**, and score changes from baseline **(C)** in the two groups.

**Figure 7:**
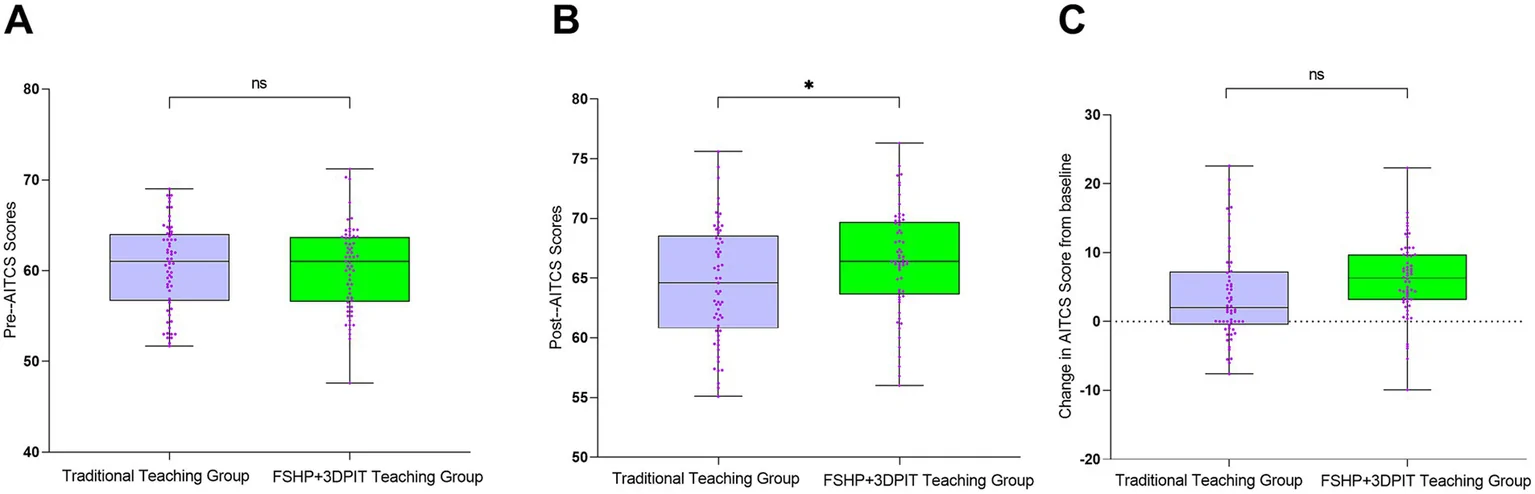
Box plots of AITCS scores at baseline **(A)**, post‑intervention **(B)**, and score changes from baseline **(C)** in the two groups.

**Figure 8:**
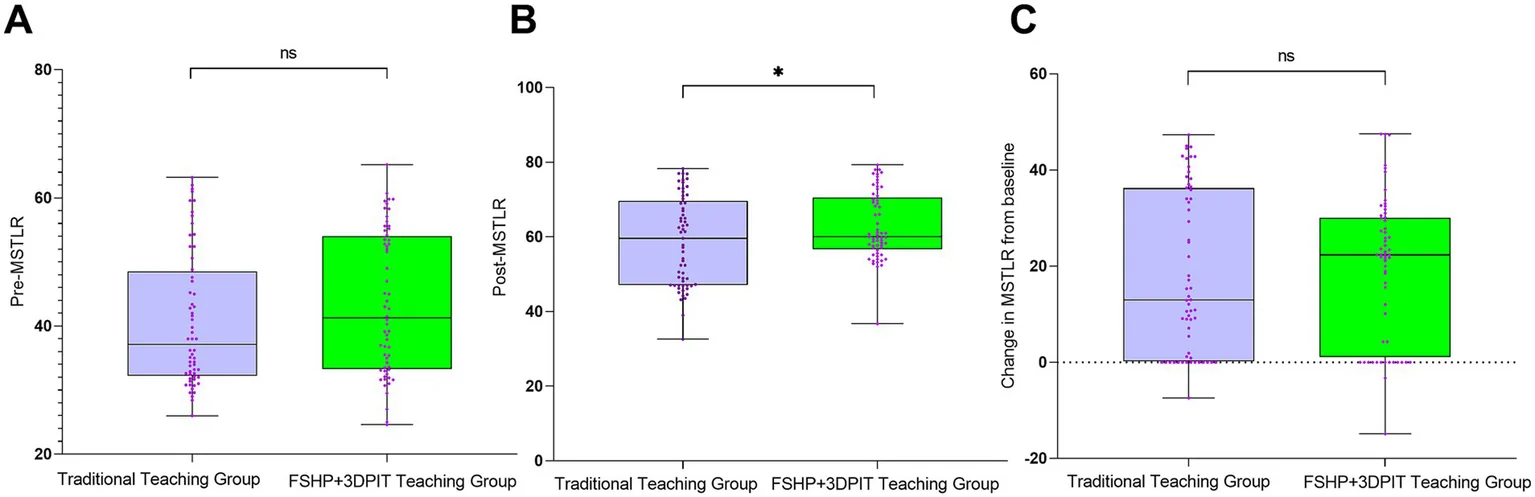
Box plots of MSTLR scores at baseline **(A)**, post‑intervention **(B)**, and score changes from baseline **(C)** in the two groups.

We first used independent sample t-tests to compare raw pretest, posttest and score change values between groups for visual presentation (Figures 3–8). Analysis of covariance (ANCOVA) adjusted for baseline scores was further adopted as the primary analytical method to eliminate the interference of initial level differences, and all adjusted statistical outcomes are summarized in Table 2.

**Table 2:** Analysis of covariance (ANCOVA) for post-intervention scores adjusted by baseline levels.

Outcome index	Adjusted mean (Observation group)	Adjusted mean (Control group)	Adjusted mean difference (95% CI)	*F* value	df	*P* value	Partial η^2^
NMLE simulation	69.87 ± 0.61	61.34 ± 0.61	8.53 (6.78–10.28)	92.48	1,117	<0.001	0.439
SMC	26.02 ± 0.37	24.89 ± 0.37	1.13 (0.21–2.05)	5.827	1,117	0.017	0.047
SDLA	117.74	117.32	0.42 (−0.53–1.37)	0.774	1,117	0.381	0.007
SEGUE	21.28 ± 0.08	21.52 ± 0.08	−0.24 (−0.36−−0.12)	15.632	1,117	<0.001	0.117
AITCS	66.54 ± 0.68	64.82 ± 0.68	1.72 (0.41–3.03)	6.854	1,117	0.01	0.055
MSTLR	60.72 ± 0.61	59.01 ± 0.61	1.71 (−1.52–4.94)	1.106	1,117	0.295	0.009

ANCOVA adjusted for baseline score of each corresponding indicator; Observation group: FSHP+3DPIT teaching model; Control group: traditional teaching model.

①At baseline, NMLE simulation scores were comparable between the observation and control groups (56.18 ± 5.39 vs. 56.21 ± 5.47; t = 0.031, df = 118, *p* = 0.975; Cohen’s d = 0.01, 95% CI: −1.83 to 1.77). After intervention, the observation group showed higher unadjusted post-test scores compared with the control group (71.02 ± 7.91 vs. 61.56 ± 9.82). Both change-score t-test and ANCOVA adjusting for baseline values demonstrated significant intergroup differences. The mean score change was 14.84 ± 9.76 in the observation group versus 5.35 ± 11.29 in the control group (*t* = 4.82, df = 118, *p*<0.001, Cohen’s d = 0.88). After baseline adjustment via ANCOVA, the adjusted post-test mean was 69.87 ± 0.61 for the observation group and 61.34 ± 0.61 for controls (mean difference = 8.53, 95% CI: 6.78–10.28; *F* = 92.48, df = 1,117, P<0.001, partial η^2^ = 0.439) (Figure 3).

②At baseline, SMC scores were comparable between the observation and control groups (25.45 ± 5.30 vs. 24.55 ± 3.71; *t* = 1.052, df = 118, *p* = 0.294; Cohen’s d = 0.19, 95% CI: −0.96 to 2.86). After intervention, the observation group showed higher unadjusted post-test scores compared with the control group (26.08 ± 4.12 vs. 24.83 ± 3.86). Both change-score t-test and ANCOVA adjusting for baseline values demonstrated significant intergroup differences. The mean score change was 0.63 ± 4.98 in the observation group versus 0.28 ± 4.21 in the control group (*t* = 2.018, df = 118, *p* = 0.0458, Cohen’s d = 0.37). After baseline adjustment via ANCOVA, the adjusted post-test mean was 26.02 ± 0.37 for the observation group and 24.89 ± 0.37 for controls (mean difference = 1.13, 95% CI: 0.21–2.05; *F* = 5.827, df = 1,117, *p* = 0.017, partial η^2^ = 0.047) (Figure 4).

③At baseline, self-directed learning ability (SDLA) scores were comparable between the observation and control groups (91.88 ± 16.64 vs. 92.86 ± 14.72; *t* = 0.33, df = 118, *p* = 0.7418; Cohen’s d = 0.06, 95% CI: −4.62 to 6.46). After intervention, the observation group achieved significantly higher unadjusted SDLA scores (117.80 ± 2.03 vs. 117.20 ± 2.01; *t* = 2.045, df = 118, *p* = 0.0431; Cohen’s d = 0.38, 95% CI: 0.02 to 1.28). The crude change-score t-test also showed a significant intergroup difference in score improvement (mean change: 25.92 ± 15.01 vs. 24.34 ± 13.99; *t* = 2.187, df = 118, *p* = 0.0307; Cohen’s d = 0.39, 95% CI: 0.306 to 6.170). However, after baseline adjustment via analysis of covariance (ANCOVA), the intergroup difference was no longer statistically significant. The adjusted post-test mean was 117.74 for the observation group and 117.32 for controls (mean difference = 0.42, 95% CI: −0.53 to 1.37; *F* = 0.774, df = 1,117, *p* = 0.381, partial η^2^ = 0.007) (Figure 5).

④At baseline, SEGUE scores were comparable between the observation and control groups (17.24 ± 0.57 vs. 17.05 ± 0.55; *t* = 1.836, df = 118, *p* = 0.069; Cohen’s d = 0.34, 95% CI, −0.01 to 0.39). After intervention, the observation group showed higher unadjusted post-test scores compared with the control group (21.54 ± 0.60 vs. 21.26 ± 0.63). Both change-score t-test and ANCOVA adjusting for baseline values demonstrated significant intergroup differences. The mean score change was 4.49 ± 0.68 in the observation group versus 4.02 ± 0.71 in the control group (*t* = 2.351, df = 118, *p* = 0.020, Cohen’s d = 0.43). After baseline adjustment via ANCOVA, the adjusted post-test mean was 21.52 ± 0.08 for the observation group and 21.28 ± 0.08 for controls (mean difference = 0.24, 95% CI, 0.12–0.36; *F* = 15.632, df = 1,117, *p* < 0.001, partial η^2^ = 0.117) (Figure 6).

⑤At baseline, AITCS scores were comparable between the FSHP+3DPIT teaching group and the traditional teaching group (64.61 ± 5.23 vs. 64.48 ± 5.19; t = 0.132, df = 118, *p* = 0.895; Cohen’s d = 0.02, 95% CI: −1.82 to 2.08). After intervention, the FSHP+3DPIT teaching group showed higher unadjusted post-test scores compared with the traditional teaching group (66.57 ± 5.31 vs. 64.79 ± 5.27). The change-score t-test demonstrated no significant intergroup difference in score elevation (*t* = 0.207, df = 118, *p* = 0.836, Cohen’s d = 0.04). Analysis of covariance adjusting for baseline AITCS values demonstrated significant intergroup differences. After baseline adjustment via ANCOVA, the adjusted post-test mean was 66.54 ± 0.68 for the FSHP+3DPIT teaching group and 64.82 ± 0.68 for the traditional teaching group (mean difference = 1.72, 95% CI: 0.41–3.03; *F* = 6.854, df = 1,117, *p* = 0.010, partial η^2^ = 0.055) (Figure 7).

⑥At baseline, transformative learning ability (MSTLR) scores were comparable between the observation and control groups (40.81 ± 10.22 vs. 43.46 ± 11.28; *t* = 1.340, df = 118, *p* = 0.1828; Cohen’s d = 0.24, 95% CI: −1.267 to 6.570). After intervention, the observation group achieved significantly higher MSTLR raw scores (58.73 ± 13.45 vs. 62.88 ± 18.02; *t* = 2.176, df = 118, *p* = 0.0315; Cohen’s d = 0.39, 95% CI: 0.3746 to 7.937). The within-group improvement (change-from-baseline value) showed no statistical difference between two groups (mean change: 18.25 ± 12.84 vs. 19.45 ± 15.37; *t* = 0.4157, df = 118, *p* = 0.6784; Cohen’s d = 0.08, 95% CI: −4.541 to 6.955). Analysis of covariance adjusting for baseline MSTLR values revealed no significant intergroup difference. After baseline adjustment via ANCOVA, the adjusted post-test mean was 60.72 ± 0.61 for the observation group and 59.01 ± 0.61 for controls (mean difference = 1.71, 95% CI: −1.52 to 4.94; *F* = 1.106, df = 1,117, *p* = 0.295, partial η^2^ = 0.009) (Figure 8).

## 4 Discussion

### 4.1 Interpretation of core research results

This study found that the 3D printing-assisted family-school-hospital collaborative teaching model was associated with greater improvements in interns’ mastery of urinary tumor screening knowledge in knowledge related to urinary system tumor early screening. It also exerted positive effects on their social medical curiosity, self-directed learning ability and doctor-patient communication skills.

Although the raw post-intervention scores of inter-professional teamwork competence and transformative learning readiness were higher in the observation group, the statistically significant between-group difference disappeared after adjusting for baseline change scores. This non-significant result cannot be attributed to baseline imbalance, as Table 1 confirmed no inter-group differences in the two indicators at baseline (all *p* > 0.05). Several potential reasons are explained as follows.

First, inter-professional teamwork and transformative learning readiness belong to long-term comprehensive core competencies of medical students, which rely on cumulative clinical practice over years. The 6-month short-term intervention in this study was insufficient to produce stable, lasting inter-group gaps.

Second, the family-school-hospital collaborative model mainly focuses on urinary tumor screening knowledge and individual self-directed learning, while it lacks targeted group training modules for multidisciplinary cooperation and transformative practice. The intervention design has limited targeted promotion effect on these two abilities.

Third, individual differences in interns’ previous clinical rotation experience and learning initiative diluted the intervention effect after baseline correction.

In subsequent research, we will extend the intervention cycle and add special teamwork and transformative learning training sessions to further explore the model’s effect on these two dimensions. The above findings are consistent with previous studies confirming that 3D visualization optimizes anatomical learning outcomes and family-involved interventions facilitate knowledge application (10, 11, 25, 26).

3D printed models restore the anatomical structures and pathological features of urinary organs vividly, making up for the limitations of conventional two-dimensional teaching. This helps interns identify disease manifestations and master key points of screening, diagnosis and treatment. Supported by the WeChat-based family-school-hospital platform, interns transformed theoretical knowledge into practical skills including health education and screening guidance under tutor supervision. The model alleviates the disconnection between learning and practice existing in traditional medical education, and enables interns to participate in family tumor prevention work during internship.

### 4.2 Core innovations

This study puts forward three improvements to address existing research gaps in tumor secondary prevention and medical education.

First, this research adopts family WeChat groups as the intervention carrier. This platform features high penetration, easy access and strong interpersonal trust, which differ from social tools widely used in Western countries (4). Combined with tumor early screening practice, it mobilizes medical students and clinical staff to supplement the limited screening capacity of community medical workers (3). The proposed mode adapts to local conditions and provides a practical reference for tumor prevention practice (27, 28).

Second, this study constructs an integrated competency system for tumor early screening, covering social medical curiosity, self-directed learning, doctor-patient communication, inter-professional teamwork and transformative learning readiness. We also clarified the positioning of each ability in practical work. Although the intervention did not produce significant effects on teamwork and transformative learning readiness after baseline correction, this framework can still serve as a reference for follow-up research on competency training for tumor prevention (20–24, 29).

Third, this study establishes an intervention framework that combines 3D printing technology, family-school-hospital cooperation and family WeChat groups. The integration of technical support, multi-party collaboration and practical scenarios forms a complete teaching mode for clinical interns.

### 4.3 Generalizability and extension of the research model

This study validated the intervention model with urinary system tumors as the research object. In view of the training objectives and core logic of the model, it can be adapted for the screening and prevention of other systemic malignant tumors. The competencies cultivated in this study are the basic abilities required for family-based tumor screening, and are applicable across different tumor types (20–24, 29).

With the support of mature 3D printing technology applied in chronic disease education (30), the model can be extended to visualize anatomical and pathological features of breast, cervical, lung, gastrointestinal and other tumors. Combined with family WeChat groups, interns can assist family members in recognizing early tumor signs. For example, interns can use 3D models to interpret manifestations such as breast nodules and cervical contact bleeding, and guide relatives to receive standardized screening. They can also identify non-specific symptoms including cough, hemoptysis and upper abdominal discomfort, and remind people to seek timely medical assessment (1, 2).

The extended application of this model can give full play to the role of medical interns in family health management, and help raise family health awareness for early tumor detection.

### 4.4 Research limitations and future research directions

This study obtained positive results and provides practical references for tumor secondary prevention and medical education practice. Several limitations need to be acknowledged. First, this is a single-center study with a relatively small sample size. Multi-center investigations with larger cohorts are needed to further verify the generalizability of the findings. Second, the effectiveness of the model was only verified in urinary system tumors, and its performance for other common malignancies remains to be explored. Third, the evaluation mainly focused on interns’ knowledge and comprehensive abilities. Indicators such as family members’ screening compliance and clinical outcomes were not included, so the overall practical effect cannot be fully assessed. Fourth, the 6-month intervention only reflected short-term effects. Long-term follow-up is required to observe whether the acquired competencies can be maintained after graduation and the sustained application effect of the model. Fifth, this study did not track family members’ screening behaviors and health changes, which are important indicators to evaluate the practical value of the intervention.

Based on the above limitations, relevant follow-up work can be carried out in the following directions. First, conduct multi-center and large-sample studies to further assess the applicability of this combined intervention model. Second, extend the model to lung cancer, breast cancer, cervical cancer, gastrointestinal cancer and other common tumors, and explore targeted optimization strategies for different scenarios. Third, improve the evaluation system by adding objective indicators such as family screening compliance and early diagnosis rate, and adopt qualitative methods like interviews to achieve comprehensive assessment. Fourth, explore cooperation with community health institutions to improve the whole workflow of tumor screening and prevention. Fifth, carry out long-term follow-up to monitor the sustainability of interns’ abilities and the continuous dissemination of screening knowledge, so as to accumulate evidence for further application of the model.

### 4.5 Theoretical scientificity and multimodal early screening practice value of the study design

The 3D printing-assisted family-school-hospital intervention model for urinary system tumor early screening is supported by classic theories in medical education and public health, and matches current multimodal practice in this field, which proves the rationality and feasibility of the research design.

First, Bandura’s self-efficacy theory is the core theoretical basis of this research (31). Three-dimensional printed models restore anatomical structures and early pathological changes of urinary system lesions, helping interns build intuitive cognition. Practical activities including health education and family risk assessment on WeChat groups enable interns to accumulate practical experience and improve their self-efficacy in tumor screening and health guidance. These activities also enhance families’ willingness to participate in screening, and promote the transformation of theoretical knowledge into practical behaviors (32).

Second, the multi-subject collaborative design conforms to the connotation of interprofessional education and cooperative learning theory. With the joint participation of clinical tutors, technical staff and family participants, the model facilitates the delivery and application of tumor screening knowledge. It also creates practical scenarios to cultivate interns’ teamwork abilities, which is in line with the practice-oriented development trend of modern medical education.

In addition, the intervention design keeps pace with the progress of urinary tumor early screening. Emerging non-invasive detection techniques and molecular markers such as urinary exosomes and miRNAs show application potential in household screening scenarios (33). Advanced imaging strategies including ^68^Ga-PSMA PET/CT and multiparametric MRI greatly elevate the detection efficiency of early clinically significant prostate cancer, providing solid evidence for standardized urinary tumor screening training (34). 3D visualization technology compensates for the shortcomings of traditional methods in anatomical recognition and lesion localization. The combination of the above multi-dimensional techniques forms an integrated working mode (35). This study provides a referable exploration for tumor prevention work, and offers ideas for follow-up studies covering more tumor types and family-based scenarios.

### 4.6 Limitations

This study has several limitations:

1. This quasi-experimental non-randomized grouping based on interns’ boarding status introduces inherent selection bias. Although we balanced multiple measurable confounding factors at baseline, unmeasured variables such as individual self-learning initiative, family health attention degree and long-term clinical learning habits cannot be fully eliminated. Therefore, all results of this study only demonstrate statistical associations between the combined teaching model and score improvement, and we cannot draw definitive causal conclusions that the intervention independently improves trainees’ core competencies. Limited to 120 valid participants, advanced confounding adjustment methods including propensity score matching and multivariate regression cannot be implemented to further eliminate bias. Subsequent large-sample multi-center randomized controlled trials are required to verify the causal effect of this model.
2. The intervention group adopted an integrated multi-component teaching scheme combining 3D printing models, WeChat remote tutoring, one-on-one mentor guidance and family-participated health education. This study cannot separate the independent contribution of each single module to score improvement, and follow-up segmented intervention research is needed to identify core effective components.
3. Due to the non-randomized grouping design based on students’ residence and after-class availability to fit real-world internship management, potential selection bias may exist.
4. All evaluation indicators rely on self-reported scales, which may bring subjective response bias. Subsequent research can integrate objective clinical performance evaluation and qualitative interview data to realize multi-dimensional assessment of students’ core competencies.
5. The intervention required additional after-class time for participation in extra teaching activities, which may influence student compliance.
6. The limited sample size precluded subgroup analysis by gender, educational background, or career intention.
7. The 6-month intervention reflects only short-term effects; long-term follow-up is needed to evaluate sustained improvements.
8. This research only evaluates interns’ theoretical examination and self-reported scale scores, without tracking objective clinical indicators including urinary tumor screening referral rate, early tumor detection rate and family screening compliance. We cannot confirm whether the score improvement can be transformed into real-world secondary cancer prevention benefits.

## 5 Conclusion

This study indicates that the 3D printing-assisted, family WeChat group-based family-school-hospital collaborative intervention is associated with meaningful improvements in medical students’ urinary tumor screening knowledge and partial core competencies of urinary system tumor early screening and their core competencies, including social medical curiosity, self-directed learning ability, and doctor-patient communication. This model makes full use of family-level medical resources and provides a feasible, low-cost, and sustainable strategy for tumor secondary prevention in China. The approach can also be extended to other tumors and populations, helping to achieve the deep integration of medical education and public health practice.

## Data Availability

The raw data supporting the conclusions of this article will be made available by the authors, without undue reservation.
