# Supplementary material for: Family-school-hospital collaboration model for urinary system tumor early screening education
Source: Front Public Health. 2026 Aug 26;14:1837667. doi: 10.3389/fpubh.2026.1837667 (PMC13553895; doi:10.3389/fpubh.2026.1837667)
Supplement: Supplementary file 1 [file Data_Sheet_1.PDF]

Supplementary Material: Scales Used in This Study

The following validated scales were used in this study, as shown in Figure 6 to Figure 10 in the main manuscript. The corresponding references are listed below.

Figure 6 (Medical Curiosity Scale)

Yang TT, Wang Y, Huang ZJ, et al. Sinicization and psychometric validation of the Medical Curiosity Scale among undergraduate nursing students. Journal of Nursing Science, 2025, 40(8): 75-78. (In Chinese)

Figure 7 (Self-directed Learning Ability Assessment Scale)

Wang XD, Tang GQ, Wang SZ, et al. Construction of a self-directed learning ability assessment scale for medical students. Chinese Journal of Health Psychology, 2014, 22(7): 1034-1037. (In Chinese)

Figure 8 (SEGUE Doctor-Patient Communication Scale)

Shen L, Sun G. Evaluation of doctors' doctor-patient communication skills based on the SEGUE scale. Chinese General Practice, 2017, 20(16): 1998-2002. (In Chinese)

Figure 9 (Simplified AITCS Scale)

Cui J, Chen XP, Lao YW, Zhuang YY. Chinese version and psychometric validation of the simplified Assessment Instrument for Teamwork Competence Scale (AITCS). Nursing and Rehabilitation Journal, 2019, 18(9): 1-6. (In Chinese)

Figure 10 (Transformative Learning Readiness Scale for Medical Students)

He JQ, Yan WX, Deng RZ, et al. Development and psychometric validation of the Transformative Learning Readiness Scale for medical students. Chinese Journal of Higher Medical Education, 2025(8): 43-46. (In Chinese)

The detailed English items and scoring criteria of each scale are shown in the following tables.

Fig6：Yang TT, Wang Y, Huang ZJ, et al. Sinicization and psychometric validation of the Medical Curiosity Scale among undergraduate nursing students [J]. J Nurs Sci, 2025, 40(8): 75-78. (In Chinese)

Fig7:Wang XD, Tang GQ, Wang SZ, Ma JD, Liu W, Tian H, Gao YS. Construction of a self-directed learning ability assessment scale for medical students [J]. Chin J Health Psychol, 2014, 22(7): 1034-1037. (In Chinese)

Fig8：Shen LJ, Sun G. Evaluation of doctors' doctor-patient communication skills based on the SEGUE scale [J]. Chin Gen Pract, 2017, 20(16): 1998-2002. (In Chinese)32. LI SJ, YU HP, CHEN LP, PENG YQ. Sinicization and psychometric validation of the Interprofessional Team Collaboration Practice Assessment Scale [J]. Chinese Journal of Modern Nursing, 2021, 27(33): 4512-4518. (in Chinese)

Fig9：Cui J, Chen XP, Lao YW, Zhuang YY. Chinese version and psychometric validation of the simplified Assessment Instrument for Teamwork Competence Scale (AITCS). Nursing and Rehabilitation Journal. 2019; 18(9): 1-6. (in Chinese)

Fig10：He JQ, Yan WX, Deng RZ, Chen SQ, Dong LJ, Ma X, Huang Q, Ao B. Development and psychometric validation of the Transformative Learning Readiness Scale for medical students [J]. Chin J Higher Med Educ, 2025(8): 43-46. (In Chinese)

Fig6. Social Curiosity Scale (7-point Scoring Criteria)

(Referenced the localized Medical Curiosity Scale by Yang Tiantian, which is derived from the Social Curiosity Scale of Heidelberg University. This scale specifically measures social curiosity, which was found to be significantly improved after the SFPC+3DIPT intervention, while intellectual curiosity showed no significant change.)

Scoring Grade	English Scoring Standard	Core Reference Direction of Social Curiosity
1	Strongly Disagree	No interest in patients' social background and doctor-patient/doctor-doctor social information
2	Disagree Greatly	Almost no willingness to explore relevant social information
3	Disagree Moderately	Low interest in relevant social information<br>unwilling to take the initiative to understand
4	Neutral/Uncertain	Neutral interest in relevant social information<br>no clear exploration tendency
5	Agree Moderately	Certain interest in relevant social information<br>willing to understand passively
6	Agree Greatly	High interest in relevant social information<br>take the initiative to understand
7	Strongly Agree	Strong interest in relevant social information<br>take active initiative to explore

Reliability: Cronbach's α coefficient of the scale was 0.852, indicating good internal consistency.

Fig 7. Scoring Criteria of Self-Directed Learning Ability Scale for Medical Students

This scale was developed by Wang Xiaodan et al., which is suitable for quantitative evaluation of medical students' self-directed learning ability. This scale was significantly improved after the SFPC+3DIPT intervention.

Scoring Grade	English Scoring Standard	Core Interpretation of Self-Directed Learning Ability
1	Completely Inconsistent	No willingness to set learning goals, implement plans, utilize resources, or reflect on effects; passive in learning.
2	Basically Inconsistent	Rarely takes initiative in goal-setting, planning, resource use, or reflection; mostly follows others' arrangements.
3	Neutral/Uncertain	Occasional initiative in learning behaviors; no consistent pattern of goal-setting, planning, or reflection.
4	Basically Consistent	Frequently takes initiative to set goals, implement plans, utilize resources, and reflect on effects; shows clear learning initiative.
5	Completely Consistent	Consistently demonstrates strong initiative in all aspects: clear goal-setting, proactive plan implementation, effective resource utilization, and regular reflective evaluation of learning outcomes.

This scale was developed by Wang Xiaodan et al., consisting of 6 factors and 30 items with a 5-point Likert scoring system. The total score ranges from 30 to 150, with a higher score indicating a stronger self-directed learning ability.

Fig 8. Scoring Criteria of Doctor-Patient Communication Skills (SEGUE Framework, Binary Scoring)

This scale is the Chinese version of the SEGUE framework, revised and localized by China Medical University, adapted from the original scale developed by Gregory Makoul et al. in 2001.

Scoring Grade	English Scoring Standard	Core Reference Direction of Doctor-Patient Communication Skills
**0**	**No**	**Failed to complete the corresponding doctor-patient communication behavior/item**
**1**	**Yes**	**Successfully completed the corresponding doctor-patient communication behavior/item**

Supplementary Notes:

1. Scale Structure: A total of 25 items, divided into 5 dimensions: Setting the stage (S), Eliciting information (E), Giving information (G), Understanding the patient's perspective (U), Ending the encounter (E).

2. Scoring Rules: Binary scoring (0 = not achieved, 1 = achieved) was adopted; the total score is the sum of all item scores.

3. Score Range & Interpretation: Total score ranges from 0 to 25 points. Higher scores indicate stronger doctor-patient communication skills (the median score of Chinese medical students/interns is 16 points, and the post-intervention mean score is approximately 18.5 points in localized studies).

4. Reliability & Validity: The intraclass correlation coefficient (ICC) of the Chinese version is 0.7185, Cronbach's α coefficient is 0.90, and the criterion validity is 0.71, showing good psychometric properties.

Fig 9. Scoring Criteria of the Simplified Assessment Instrument for Teamwork Competence Scale (AITCS, Chinese Version)

This scale is the simplified Chinese version of AITCS, developed and validated among medical staff at Sir Run Run Shaw Hospital, Zhejiang University, for assessing inter-professional teamwork competence.

Scoring Grade	English Scoring Standard	Core Reference Direction of Inter-professional Teamwork Competence
1	Strongly Disagree	Extremely low level of inter-professional teamwork competence in the corresponding dimension
2	Disagree	Low level of inter-professional teamwork competence in the corresponding dimension
3	Neutral	Moderate level of inter-professional teamwork competence in the corresponding dimension
4	Agree	High level of inter-professional teamwork competence in the corresponding dimension
5	Strongly Agree	Extremely high level of inter-professional teamwork competence in the corresponding dimension

Supplementary Notes

1. Scale Structure: A total of 23 items, divided into 3 dimensions: Partnership, Team Coordination, and Team Collaboration.

2. Scoring Rules: A 5-point Likert scale was adopted (1 = strongly disagree, 5 = strongly agree); the total score is the sum of all item scores.

3. Score Range & Interpretation: Total score ranges from 23 to 115 points; the actual score of medical students/interns is concentrated in 50-75 points (higher scores

indicate stronger interprofessional teamwork competence).

4. Reliability & Validity: According to the validation study at Sir Run Run Shaw Hospital, the Cronbach's α coefficient of the total scale is 0.912, the split-half reliability was 0.833, and the test-retest reliability was 0.720, showing good psychometric properties.

Fig 10. Scoring Criteria of the Medical Student Transformative Learning Readiness (MSTLR) Scale for Medical Students

Dimension	Number of items	Scoring Method	Reverse Scoring Items	Total Score Range	Score Interpretation
Environmental Perception	7	5-point Likert scale (1-5)	None	16~80 points	Higher scores indicate a higher level of transformative learning readiness
Self-Reflection	6	1=Strongly disagree, 5=Strongly agree	11,12		
Outward Exploration	3		None		
Total Scale	16		11,12	16~80 points	Higher scores = better readiness

The overall Cronbach's α coefficient of the scale was 0.887, and the Cronbach's α coefficients of each dimension ranged from 0.773 to 0.859, showing good reliability and validity.

- This scale can effectively evaluate the readiness of medical students to transform theoretical knowledge into practical ability.
